# There’s just not enough time: a mixed methods pilot study of hepatitis C virus screening among baby boomers in primary care

**DOI:** 10.1186/s12875-020-01327-2

**Published:** 2020-12-02

**Authors:** Monica L. Kasting, Julie Rathwell, Kaitlyn M. Gabhart, Jennifer Garcia, Richard G. Roetzheim, Olveen Carrasquillo, Anna R. Giuliano, Susan T. Vadaparampil

**Affiliations:** 1 Department of Public Health, Purdue University, 812 W. State Street, West Lafayette, IN 47907 USA; 2 Center for Immunization and Infection Research in Cancer, H. Lee Moffitt Cancer Center, 12902 Magnolia Drive, Tampa, FL 33612 USA; 3 Department of Cancer Epidemiology, H. Lee Moffitt Cancer Center, 12902 Magnolia Drive, Tampa, FL 33612 USA; 4 Department of Health Outcomes and Behavior, H. Lee Moffitt Cancer Center, 12902 Magnolia Drive MRC-COEE, Tampa, FL 33612 USA; 5 Department of Family Medicine, University of South Florida, Tampa, USA; 6 Division of General Internal Medicine, University of Miami, 1120 NW 14th Street, Miami, FL 33136 USA

**Keywords:** Hepatitis C, Screening, Health services research, Professional practice gaps, Attitude of health personnel, Primary care

## Abstract

**Background:**

Liver cancer rates are rising and hepatitis C virus (HCV) is the primary cause. The CDC recommends a one-time HCV screening for all persons born 1945–1965 (baby boomers). However, 14% of baby boomers have been screened. Few studies have examined primary care providers’ (PCP) perspectives on barriers to HCV screening. This study examines current HCV screening practices, knowledge, barriers, and facilitators to HCV screening recommendation for baby boomers among PCPs.

**Methods:**

We conducted a mixed methods pilot study of PCPs. *Quantitative*: We surveyed PCPs from 3 large academic health systems assessing screening practices, knowledge (range:0–9), self-efficacy to identify and treat HCV (range:0–32), and barriers (range:0–10). *Qualitative*: We conducted interviews assessing patient, provider, and clinic-level barriers to HCV screening for baby boomers in primary care. Interviews were audio recorded, transcribed, and analyzed with content analysis.

**Results:**

The study sample consisted of 31 PCPs (22 survey participants and nine interview participants). All PCPs were aware of the birth cohort screening recommendation and survey participants reported high HCV testing recommendation, but qualitative interviews indicated other priorities may supersede recommending HCV testing. Provider knowledge of viral transmission was high, but lower for infection prevalence. While survey participants reported very few barriers to HCV screening in primary care, interview participants provided a more nuanced description of barriers such as lack of time.

**Conclusions:**

There is a need for provider education on both HCV treatment as well as how to effectively recommend HCV screening for their patients. As HCV screening guidelines continue to expand to a larger segment of the primary care population, it is important to understand ways to improve HCV screening in primary care.

**Supplementary Information:**

The online version contains supplementary material available at 10.1186/s12875-020-01327-2.

## Background

Hepatocellular carcinoma (HCC) is one of the few cancers that has increased in incidence and mortality over the last decade in the United States [1]. Approximately half of all U.S. cases are caused by chronic hepatitis C virus (HCV) infection [2, 3]. Between 2.7 to 3.9 million people in the U.S. are currently infected with HCV [4] and thereby at risk for serious health sequelae, including end stage liver disease and HCC [5]. Direct-acting antivirals cure ~ 90% of patients infected with HCV [6] and reduce cancer risk by 50% [2]. However, 50–75% of those with HCV are unaware they are infected [7, 8]. Without intervention, HCV-associated disease will increase and peak in 2030 [9].

Individuals born 1945–1965 [10] have nearly five times the prevalence of HCV infection compared to other birth cohorts [11]. In 2012, the Centers for Disease Control and Prevention (CDC) augmented their risk-based HCV screening guidelines to include a one-time screening for persons born 1945–1965 (i.e., baby boomers) [12]; followed by the U.S. Preventive Services Task Force (USPSTF) in 2013 [13]. However, according to the 2016 National Health Interview Survey (NHIS), only 14.1% of adults in this age group reported ever being screened for HCV [14]. Furthermore, in 2020 the USPSTF expanded the recommendation to include a one-time screening for all persons ages 18–79 [15], further highlighting the need to explore barriers to HCV screening in primary care.

Healthcare provider recommendation is a significant predictor of preventive health behaviors, such as screening [16, 17]. Yet, the opportunity to recommend screening is often missed during a clinic visit [18, 19]. Providers face competing demands during medical encounters, which may influence how preventive services are recommended and provided. The Competing Demands Model [20], proposes three domains influencing providers’ recommendations of preventive health services: provider factors (e.g. personal characteristics, knowledge, beliefs), patient factors (e.g. type of visit, demographic characteristics), and practice factors (e.g. reminder systems, standing orders) [20]. Using the Competing Demands Model as a theoretical framework, we conducted a mixed-methods pilot study to examine current HCV screening practices, knowledge, barriers, and facilitators to HCV screening recommendation for baby boomers among primary care providers (PCPs) in clinics with documented low rates of HCV screening [21, 22].

## Methods

This mixed-methods pilot study used convergent parallel design procedures [23] and consisted of a survey and qualitative interviews as a preliminary step to assess barriers to HCV screening. Given the pilot and exploratory nature of the study, we worked with clinics to implement our study in a manner that was most convenient and least disruptive to their clinic workflow. These varied approaches are detailed below. The study was approved by the scientific review committee at the Moffitt Cancer Center, and by the institutional review boards at Purdue University and the University of Florida.

### Survey participant recruitment

The survey consisted of a convenience sample of PCPs from three academic health centers in Florida: the University of South Florida (USF), the University of Florida (UF), and the University of Miami (UM). Recruitment strategies were based on the preferences of the clinical leadership of each institution. At USF and UM, we extracted publicly available healthcare provider email addresses for any healthcare provider listed as an adult family medicine or internal medicine physician, nurse practitioner, or physician assistant. Recruitment at UF occurred via purposive sampling strategies. Identified eligible PCPs were sent an introductory e-mail describing the study and requesting their participation in an online survey. Inclusion criteria for survey providers were: 1) a PCP in family medicine or internal medicine at USF, UF, or UM, and 2) delivers care for patients born 1945–1965. The survey link was provided in the email. Additionally, providers could opt-out of the survey to avoid future reminder contacts. Upon completion, providers were automatically directed to a separate electronic form to provide a mailing address to receive the study incentive ($50 gift card). Those who did not opt out or complete the survey after the initial email were sent a reminder email at two and/or 4 weeks.

### Survey instrument

The 24-item survey took approximately 5 min to complete. Previously validated scales were used when available, and adapted to fit the study population.

#### General Screening Practices and Reminders

General HCV-related questions include the PCPs’ personal screening practices, birth cohort recommendation awareness, and HCV-related reminders [24–26]. HCV recommendation practices assessed included: 1) strength (i.e. how strongly they recommend HCV screening to their baby boomer patients) on a 5-point scale from “I strongly recommend” to “I recommend against;” 2) consistency (i.e. how often they recommend their baby boomer patients get screened) on a 5-point scale from never/almost never (approximately 10% of the time) to always/almost always (greater than 90% of the time); and 3) presentation (i.e. presenting HCV screening as routine vs. optional) were measured using previously validated questions, adapted from the literature on human papillomavirus vaccination [27–30].

#### Knowledge (9-items; range 0–9)

PCPs answered 10 true/false HCV-related knowledge questions. One knowledge question was discarded due to ambiguous wording, resulting in a 9-item knowledge scale. PCPs had the option of replying “unsure” to any given question. Respondents were given one point for each question they answered correctly, and no points if they answered incorrectly or indicated they were unsure of the answer.

#### Provider Self-Efficacy (8-items; range 0–32)

Provider self-efficacy was measured using a previously validated measure [24]. PCPs rated their ability on a scale of 0 (none) to 4 (expert), to do the following for their patients: identify those who should be screened for HCV, discuss HCV infection and screening, and treat HCV-infected patients, among others.

#### Barriers (6-items; range 6–30)

Providers responded to 6 questions regarding barriers for both the provider and the patient on a 5-point Likert scale from strongly disagree to strongly agree. For example, a provider-level barrier question included “I do not have time to discuss HCV screening with my patients.” A question such as “My patients do not have insurance to cover the cost of HCV screening” assessed patient-level barriers.”

### Interview participant recruitment and interview procedures

Qualitative data collection and analyses align with the COnsolidated criteria for REporting Qualitative research (COREQ) checklist [31]. One institution (USF) allowed us to conduct qualitative interviews with providers in addition to the surveys. We conducted these interviews using a purposive sample of healthcare providers who were a primary care provider at the USF Family Medicine Clinic, delivered care for patients born 1945–1965, and were not part of the survey sample. We identified providers through the USF Health provider directory and extracted any PCP listed as adult family medicine. Eligible PCPs were sent an introductory e-mail describing the study and requesting their participation in an in-person or telephone interview.

Providers who expressed interest and completed a consent were scheduled for an in-person or phone interview. The interview guide was based on the Competing Demands Model and literature on barriers to HCV uptake. The 30–45 min interview began with over-arching questions such as “What is the current approach to HCV screening in your practice?” Participants received a $100 gift card for participating. We conducted interviews until saturation was reached (9 interviews). Prior qualitative studies on competing demands in primary care suggest that 7 interviews are needed to reach saturation (i.e., no new information with additional interviews) [32, 33].

### Data analysis

For this descriptive pilot study, the survey data, frequencies and percentages were calculated for the variables of interest to describe the population and identify areas for future study. Data from qualitative interviews were used to clarify the quantitative results. Interviews were audio-recorded, transcribed, and analyzed using inductive content analysis [34, 35]. This process involves open coding, which begins with two team members independently reading the transcripts and inductively generating themes or topics from the data. After independently coding the interviews, the two team members met, reviewed codes, and areas of disagreement are resolved through discussion. The inductive, iterative process continues by applying the themes to subsequently read data and revising [34]. We then compared and contrasted the results from the quantitative and qualitative data.

## Results

The study sample consisted of 31 PCPs (22 survey participants and 9 interview participants). Of the survey participants, 15 were from UF, 3 were from UM, and 4 were from USF. All interview participants were from USF. The majority White/Caucasian (*n* = 22; 71%), female (*n* = 18; 58%), and listed their primary clinical specialty as family medicine (*n* = 16; 52%). Mean age was 41.7 (SD = 10.6), they averaged 11.9 years practicing medicine (SD = 9.0), and 12.9% reported Hispanic ethnicity. For a full sample description, see Table 1. Table 2 depicts each study construct with columns to compare and contrast the results from the surveys and interviews.

**Table 1:** Sample description (*n* = 31)^a^

	Total Sample (*n* = 31) n (%)	Quantitative Survey Participants (*n* = 22) n (%)	Qualitative Interview Participants (*n* = 9) n (%)
Age (Mean [Range])	41.7 (29–67)	42.3 (29–65)	40.2 (29–67)
Gender			
*Male*	12 (38.7)	8 (36.4)	4 (44.4)
*Female*	18 (58.1)	13 (59.1)	5 (55.6)
Race/Ethnicity			
*White/Caucasian*	22 (71.0)	16 (72.7)	6 (66.7)
*Black/African American*	1 (3.2)	1 (4.5)	0 (0.0)
*Asian*	4 (12.9)	3 (13.6)	1 (11.1)
*Other/Prefer not to answer*	3 (9.7)	1 (4.5)	2 (22.2)
Ethnicity			
*Non-Hispanic*	26 (83.9)	18 (81.8)	8 (88.9)
*Hispanic*	4 (12.9)	3 (13.6)	1 (11.1)
Years Practicing Medicine (Mean [Range])	11.9 (1–36)	12.3 (3–35)	11.0 (1–36)
Clinic Specialty			
*Family Medicine*	16 (51.6)	9 (40.9)	7 (77.8)
*Internal Medicine*	14 (45.2)	12 (54.5)	2 (22.2)
*Internal Medicine/Pediatrics*	1 (3.2)	1 (4.5)	0 (0.0)

^a^Due to missing data, not all variables add up to 100%

**Table 2:** Comparing and contrasting quantitative and qualitative results

**Construct**	**Quantitative Findings (*n*** **= 22)**		**Qualitative Findings (*n*** **= 9)**
**Screening practices**	Recommendation awareness		“I mean, there’s so much to cover in every primary care doctor visit. …**Sometimes there’s just not enough time** to introduce the idea of hepatitis C screening and why we recommend it.” “For [baby boomer] patients without risk factors, ideally, we’ll have a conversation about a one-time screening for hepatitis C. At least that’s our goal. I think sometimes, **things get busy, time runs low, and that then maybe gets deprioritized**.” “We order [HCV screening] and we tell the patient, ‘You know **you’re due for Hepatitis C screening** because you were born between this year and this year, and you’re high-risk.’ If they’re agreeable to it, then we just order the lab and have it done.” “Generally using the rule of thumb between people that were **born between 1945 and 1965**, if they had **illicit drug use** or **injectable drug use**, if they were giving any **blood transfusions before about 1990**. Obviously if they have **acute elevations of liver enzymes**, I’m definitely screening for all hepatitis during that point. If there’s a **needle stick**, then absolutely screen for all hepatitis at that point.”
	*No*	3 (13.6)	
	*Yes*	19 (86.4)	
	Recommendation strength		
	*Strongly recommends*	16 (72.7)	
	*Recommends, but not strongly*	6 (27.3)	
	*Makes no recommendation for or against*	0 (0.0)	
	*Recommends against*	0 (0.0)	
	Personal screening practices		
	*Rarely screens patients*	0 (0.0)	
	*Screens patients with behavioral risk factors (*e.g. *injection drug use)*	15 (68.2)	
	*Screens patients when clinically indicated (*e.g. *elevated ALT)*	18 (81.8)	
	*Screens patients with age-based risk factors (*e.g. *baby boomers)*	22 (100.0)	
	Screening presentation		
	*Screening is routine*	22 (100.0)	
	*Screening is optional*	0 (0.0)	
	*I do not discuss screening with baby boomer patients*	0 (0.0)	
	Consistency of recommendation		
	*Occasionally (10–39% of the time)*	2 (9.1)	
	*About half of the time (40–59% of the time)*	2 (9.1)	
	*Usually (60–90% of the time)*	11 (50.0)	
	*Always/almost always (greater than 90% of the time)*	6 (27.3)	
**Knowledge (% correct)**	You can get HCV from a blood transfusion from an infected donor (true)	22 (100.0)	“I know basics about hepatitis C but **I’m not comfortable talking about prognosis or individual screening or staging**. … I’m going to refer them to a gastroenterologist and have them take it from there.” “It’s … **not something that I’ve learned, not something I’m comfortable with**, so I typically do just refer them to GI, and they take care of it.”
	You can get HCV by having sex with someone infected with HCV (true)	20 (90.9)	
	Perinatal transmission is not possible (false)	18 (81.8)	
	HCV can be transmitted through contaminated needles (true)	22 (100.0)	
	HCV can be contracted through injection drug use (true)	22 (100.0)	
	People who report risk behaviors should be screened yearly for HCV (true)	21 (95.5)	
	The CDC and USPSTF recommend HCV screening for baby boomers *only if* they report a behavioral risk factor (false)	22 (100.0)	
	Available curative treatments for HCV have substantial side effects (false)	16 (72.7)	
	Approximately 1 in 30 baby boomers is currently infected with HCV (true)	10 (45.5)	
	Total score (mean[SD]; Range: 0–9)	7.86 (0.94)	
**Provider Self-Efficacy**	How would you rate your proficiency in the following areas…(mean score from 0 [none] to 4 [expert])		“There are **two of us that do the treatment. So, the other ones – they would refer them to us…for** a couple visits during the Hep C treatment.” “Generally what **I’ll do is provide a referral** to see a GI specialist. I might **order additional testing** that I would anticipate the GI doctor would want, and then **checking for vaccination status of hepatitis A and B.**”
	*Ability to identify patients who should be screened*	2.6 (0.67)	
	*Ability to discuss HCV infection with patients*	2.5 (0.60)	
	*Ability to adequately refer patients to proper specialist for care*	3.0 (0.72)	
	*Ability to execute the proper next steps should a patients screen positive for HCV*	2.8 (0.81)	
	*Ability to treat HCV-infected patients and manage side-effects*	1.1 (0.94)	
	*Ability to provide a brief alcohol screen, counseling, and referral for alcohol use treatment services*	2.4 (0.59)	
	*Ability to assess and manage substance abuse comorbidities in HCV-infected patients*	1.9 (0.71)	
	*Ability to implement in-clinic procedures for universal screening of baby boomers*	2.1 (0.89)	
	Total score (Range: 0–32)	18.4 (3.8)	
**Barriers**	Please indicate the degree to which you agree or disagree with the following statements… (mean score from 1 [strongly disagree] to 5 [strongly agree])		“Hesitation if the test is **covered by the insurance** or not. That’s their biggest concern when they’re getting blood work is **try to minimize cost and copays**” “So, the provider barrier is always **time**. So, sometimes of just like, I’ve already dealt with mammogram and colonoscopy today. That’s enough screening stuff in one visit. I’ll do this one next time type thing.” “I would say **time** is the single biggest barrier to it. Whether it’s there’s just not enough time within the office visit to have a conversation about it, or whether there’s **12 other things to address and it just escapes my mind**.” “But there’s **so many things that could be addressed** during, you know, an annual, like it could be that they maybe have come for their annual but you find that their A1C is like 11% and so you’re kind of stuck doing a different type of visit.” “Well – there’s never enough **time**. Really ... there’s so many things that need to be screened. It really depends on the patient and **their other issues**.”
	*I do not have time to discuss HCV screening with my patients*	2.0 (1.05)	
	*I am not comfortable managing my patients if they screen positive for HCV infection*	1.5 (0.68)	
	*Screening for HCV infection is a less-urgent problem for my patients, compared to their other problems*	2.7 (1.10)	
	*My patients are not interested in screening when I recommend it for them*	2.0 (0.67)	
	*My patients do not have insurance to cover the cost of HCV screening*	1.9 (0.77)	
	*The cost for HCV treatment is a barrier for my patients*	2.8 (1.30)	
	Total score (Range: 5–30)	12.9 (3.02)	

### Screening practices

The majority of survey participants indicated they were aware of the birth cohort screening recommendations (*n* = 19; 86.4%) and all reported screening their baby boomer patients for HCV. All participants indicated they recommend HCV screening for their baby boomer patients with 73% indicating they strongly recommend it and 27% indicating they recommend it, but not strongly. Likewise, 100% of survey participants presented HCV screening to their baby boomer patients as routine. Yet, when asked about whether they consistently recommend HCV screening to their baby boomer patients, 18% (*n* = 4) stated they recommend screening to their patients less than 60% of the time.

This finding was further clarified during the qualitative interviews with providers acknowledging or describing the risk-based and the birth cohort screening recommendations. While the majority of providers indicated they would ideally prefer to discuss screening with their baby boomer patients, they also noted that this discussion may be a lower priority if the patient has other comorbidities or concerns and there is limited time during a patient visit.

### Knowledge

Mean knowledge score was 7.9 (SD = 0.94) with a range of 6–9. Almost all of the providers correctly answered questions related to HCV transmission (e.g. transmission through needle sharing, intravenous drug use, or from a blood transfusion from an infected donor). Knowledge regarding HCV prevalence and treatment was lower; 55% did not know that 1 in 30 baby boomers is currently chronically infected with HCV, and 27% incorrectly indicated that currently available HCV treatments had substantial side effects.

The qualitative interviews demonstrated similar knowledge patterns. While the providers were very familiar with the screening recommendations, none described the HCV infection prevalence or discussed why screening was specifically recommended for patients born 1945–1965. Six out of the nine interviewees mentioned they screened because the practice EHR provided a reminder for screening patients born 1945–1965. In discussions regarding treatment, two-thirds expressed being uncomfortable treating patients with HCV, and would instead refer to a specialist, which may be associated with a lack of knowledge about treatment.

### Provider self-efficacy

The mean score for the 8 self-efficacy items was 18.4 and had acceptable internal reliability (Cronbach’s alpha of 0.79). On the self-efficacy questions, all of the survey participants reported they were either average or above average on all but three items including their ability to: 1) treat HCV-infected patients, 2) assess and manage substance abuse comorbidities, and 3) implement an in-clinic procedure for universal screening of baby boomers. In particular, 16 of the 22 survey participants said they either had no or limited ability to treat HCV-infected individuals.

Results from the qualitative interviews also highlighted this concept with providers indicating they would only treat an HCV-infected patient if they “absolutely had to.” Most reported they would refer an HCV-infected patient to a gastroenterologist for treatment. However, a few providers reported both experience and comfort treating HCV infections. In contrast to what providers reported in their perceived self-efficacy about having no or limited proficiency in their ability to treat HCV, none agreed with the statement “I am not comfortable managing my patients if they screen positive for HCV infection.” Our qualitative interview participants expressed a similar statement, indicating “appropriate management” is referral to GI.

### Barriers

Overall, providers reported few barriers to HCV screening in the survey responses and all of them either disagreed or strongly disagreed that patient interest in screening and insurance coverage were barriers. Mean scores for each item on the Barriers scale were between 1.5 and 2.8, with a possible range of 1–5 and lower scores indicating fewer barriers. However, in the qualitative interviews, participants frequently reported insurance coverage and cost as a patient barrier while simultaneously stating that most of their patients had insurance and they had never had insurance deny it. While participants indicated their patients’ insurance would cover the cost of HCV screening, they raised additional concerns about the cost of HCV treatment, in the event a patient tested positive. Cost of HCV treatment was a barrier reported by over one-third of survey participants (36%).

Responses to the survey and interviews were also inconsistent for the other two barriers: time and patient comorbidities. Only 10% of survey participants either agreed or strongly agreed with the statement “I do not have time to discuss HCV screening with my patients.” However, in interviews, providers frequently noted time as a major barrier and indicated other medical issues that take precedence during a time-limited clinic visit. One-third (36%) of survey participants disagreed or strongly disagreed with the statement “Screening for HCV infection is a less-urgent problem for my patients, compared to their other problems.” The qualitative interviews indicated the number of comorbidities and the time to discuss them is patient-specific and will affect whether screening is discussed.

## Discussion

This mixed methods study examined current HCV screening practices, knowledge, provider self-efficacy, and barriers to HCV screening recommendation for baby boomers among primary care providers. All providers in our sample reported they either strongly or very strongly recommend HCV screening to their baby boomer patients and recommend it as routine. Research shows a strong provider recommendation is an important predictor of preventive services uptake [16, 17]. If providers in our sample were strongly recommending HCV screening, and presenting it as routine, we would expect to have very high rates of HCV screening uptake among their patients. While we do not have specific screening rates for the patients of each of our physician participants, low rates of HCV screening have been observed nationally as well as in other research that has included these health systems [14, 21, 22, 36]. This finding may indicate providers are over-reporting their use of a strong recommendation for HCV screening. Providers may benefit from performance feedback regarding HCV screening among their patients. Other research indicates that data feedback for providers increases awareness of their own personal screening rates and, in turn, increases the frequency with which they screen their patients [37]. Alternatively, this may indicate that providers are recommending it strongly when they have the opportunity to discuss it in a patient visit, but may be constrained by short appointment times coupled with more pressing health concerns.

While survey participants reported very few barriers to HCV screening in primary care, our interviews provided a more nuanced description of some of the barriers. For example, lack of time was a predominant barrier described by interview participants, but only 10% of survey participants indicated they did not have time to discuss HCV screening with their patients. Lack of time during a primary care preventive visit is a barrier frequently reported in the literature [38]. In particular, one study found it would take an average of 7.4 h per working day for a provider to address all recommended preventive care for their patients, leaving little-to-no time to discuss comorbidities or acute illnesses [39]. Other research has found that the average length of face-to-face time during primary care appointments in the United States is approximately 15 min, resulting in limited time for discussions and forcing providers and patients to prioritize which topics they will discuss [40–42]. It is unclear why PCPs in our survey sample did not endorse time as a barrier. Other research has found physician attitudes regarding the importance of a health behavior and their reported self-efficacy in treating a disease were more strongly associated with screening for the disease than the physician’s concerns about time during a clinic visit [43]. Therefore, it is possible our qualitative interview participants were more able to describe the nuances of their lack of time, including their concern about being able to treat a patient, than the survey participants. Furthermore, we did not ask survey participants how important they felt HCV screening was for their baby boomer patients. It is possible our providers had high perceived importance of HCV screening for their baby boomer patients. Other research has found that a higher perceived importance of a preventive screening test was associated with a lower perception of a lack of time as a barrier [43].

While overall knowledge in our sample was high, providers had higher knowledge regarding HCV transmission than HCV treatment. Similarly, providers reported high overall self-efficacy, but low self-efficacy on the items regarding managing and treating HCV infection. Most qualitative interview participants expressed reluctance to treat HCV infection. While research has shown patients treated by PCPs have similar outcomes to patients treated by specialists [44, 45], PCPs generally prefer referral to a specialist [46]. There have been some interventions to increase HCV treatment in primary care, particularly in rural settings with limited access to a specialist. For example, the Extension for Community Healthcare Outcomes (ECHO), uses video-conferencing technology to treat complex diseases [47]. When applied to HCV treatment, Project ECHO reported similar rates of sustained virologic response in ECHO patients compared to those treated at an academic medical facility [48]. This finding supports the potential for successful treatment of chronic HCV infection in primary care, if providers are supported with needed tools and resources.

Another area of concern identified in our study was a general lack of awareness regarding insurance coverage for the HCV antibody test and a concern regarding cost. The HCV antibody screening test is recommended preventive care and 100% of the cost is covered for any patient with insurance, as mandated by the Patient Protection and Affordable Care Act (ACA) [49]. While none of the survey participants indicated their patients lacked insurance to cover the cost, interview participants reported cost as one of the primary barriers to HCV screening. It is possible providers in our sample were unaware of the preventive coverage mandate under the ACA. Research in other areas has found providers have low awareness of what is covered by ACA [50]. However, it is also possible that, while we specifically asked providers about the cost of the antibody screening test, they may also have been commenting on the cost of follow-up testing in the event of a positive antibody test, or even the cost of treatment. Future research should aim to understand why providers may be concerned about insurance not covering the test, despite the ACA mandate, and if these concerns were actually in regards to follow-up testing, not antibody testing.

This study is among the first mixed-methods study to examine barriers to HCV screening among baby boomers in primary care. The use of quantitative survey and qualitative interviews helped to add depth and clarity to study findings. However, results should be interpreted in light of some limitations. First, our sample was subject to selection bias and it is possible providers who chose to participate in our study had higher HCV-related knowledge and/or interest in HCV screening. In addition, because we used convenience samples, it was not always possible or appropriate to determine our study response rate or gather information on people who declined participation, limiting our ability to examine possible selection biases. Second, our sample size was small, limiting the generalizability of our survey findings. Third, our interview participants were all from the same institution, resulting in similar experiences and limiting the generalizability of the findings. Fourth, this study was conducted at academic health centers with access to specialty care. The findings of this study may not be reflective of other health settings including rural practices with limited access to gastroenterology and hepatology specialists. Fifth, particularly for interview participants, responses may be subject to social desirability bias due to the lack of anonymity.

## Conclusions

There are several areas for future research identified in this study with respect to exploring lack of concordance between providers self-reported strong recommendations for HCV screening and low screening rates, provider education on HCV treatment, and increasing providers’ self-efficacy for treating HCV. Future research should also use mixed methodological approaches to gain a comprehensive and nuanced understanding of ways to increase HCV screening in diverse primary care settings that provide preventive care. Recently, the USPSTF updated their recommendations for screening baby boomers to include a one-time universal screening for all people between the ages of 18–79 [15]. This offers new opportunities for research, as the barriers to universal screening for adults in this age range are likely to be different from barriers for baby boomers or people at high risk of infection. With this expanded age range for HCV screening, it is even more important to understand ways to improve HCV screening in primary care to decrease HCV-related morbidity and mortality.

## Supplementary Information

**Additional file 1.**

**Additional file 2.**

## Data Availability

The datasets used and/or analyzed during the current study are available from the corresponding author on reasonable request.

## Abbreviations

ACA: Patient Protection and Affordable Care Act
CDC: Centers for Disease Control and Prevention
COREQ: Consolidated criteria for Reporting Qualitative research
ECHO: Extension for Community Healthcare Outcomes
HCC: Hepatocellular Carcinoma
HCV: Hepatitis C Virus
NHIS: National Health Interview Survey
PCP: Primary Care Provider
UF: University of Florida
USF: University of South Florida
USPSTF: United States Preventive Services Task Force
UM: University of Miami
