# Supplementary material for: There’s just not enough time: a mixed methods pilot study of hepatitis C virus screening among baby boomers in primary care
Source: BMC Fam Pract. 2020 Dec 2;21:248. doi: 10.1186/s12875-020-01327-2 (PMC7713319; doi:10.1186/s12875-020-01327-2)
Supplement: Supplementary file 1 — Additional file 1. [file 12875_2020_1327_MOESM1_ESM.docx]

1. To start, could you just briefly describe your role in your current clinic?
2. What is the current approach to HCV screening in your practice?
3. Would you mind giving me a brief description of current HCV screening recommendations from national organizations?
   1. What patient-reported behaviors would result in you recommending HCV screening?
   2. Do you think your patients adequately report their HCV-related behavioral risk factors? Or are these behaviors (i.e. drug use, illicit tattoos, etc.) something patients might avoid telling you?
   3. Are there any ages you screen universally, regardless of other HCV-related risk factors?

*Patient-level barriers*

1. As you think about your patients who need to be screened for HCV (those born 1945-1954, who use injection drugs, live with someone with hepatitis, etc.) what do you think are the biggest barriers to getting them screened?
2. Are your patients who need to be screened aware of the current screening recommendations?
3. Have they heard of HCV before?
4. Is lack of insurance a problem?
5. Do the patients feel HCV screening is important for them? Especially compared to all of the other screenings that are recommended for them?

*Provider-level barriers*

1. Do you think most primary care providers are aware of current screening recommendations for HCV?
2. Do you feel comfortable talking to your patient about HCV infection?
3. Do you feel comfortable recommending HCV screening to your patients?
4. How important do you think HCV screening is compared to all of the other preventive screenings that are recommended for your baby boomer patients?
5. From your perspective, what do you think are the biggest benefits to screening your patients?
6. From your perspective, what do you think are the biggest barriers to screening your patients?
   1. Is it more difficult to order and HCV screening test than other blood tests you routinely order for your patients (e.g. cholesterol panel)?
   2. What about this screening test makes it more difficult to order (e.g. it isn’t easily accessible from the EHR, the patients are hesitant due to lack of information or common understanding about HCV)?
7. If given the option, do you think your patients would prefer an at-home finger prick screening test that they mail in? Even if it isn’t covered by insurance?
8. If given the option, do you think your patients would prefer an in-clinic rapid finger prick screening test where the results are available in approximately 20 minutes? Even if it isn’t covered by insurance?

*Practice-level barriers*

1. What reminder systems are currently in place to help you remember general preventive screenings for your patients?
   1. Is HCV screening included in this?
2. Does your clinic have adequate educational resources to promote HCV screening to your patients (is it available, etc.)?
3. Is there sufficient time during a well visit for a patient to discuss HCV screening?
4. Is there sufficient time during an acute visit to discuss HCV screening?
5. What other office staff are involved in the HCV screening process?
   1. Do you have standing orders for HCV screening?
   2. Does the nurse address HCV screening during intake and order it?
6. Do health plans provide information on who needs to be screened or use it as a quality metric?
   1. Do you think HCV screening is something that should be tracked in quality measures (like HEDIS) that drive reimbursement?
   2. Are you further motivated by a concern about malpractice should a case fail to be identified?
7. Do your patients have easy access to follow-up treatment in the event they test positive?
   1. Is there infrastructure in place where you feel comfortable with the next steps should a patient be HCV positive.

*General Questions*

1. How often do you recommend HCV screening to your patients with an exposure or behavioral risk factors for HCV?
   - Never/almost never (<10% of the time)
   - Occasionally (10-39% of the time)
   - About half the time (40-59% of the time)
   - Usually (60-90% of the time)
   - Always/almost always (>90% of the time)
2. Among those for whom you recommend HCV screening, how frequently do these patients with an exposure or behavioral risk factor actually complete the test?
   - Never/almost never (<10% of the time)
   - Occasionally (10-39% of the time)
   - About half the time (40-59% of the time)
   - Usually (60-90% of the time)
   - Always/almost always (>90% of the time)
3. How often do you recommend HCV screening to your baby boomer patients?
   - Never/almost never (<10% of the time)
   - Occasionally (10-39% of the time)
   - About half the time (40-59% of the time)
   - Usually (60-90% of the time)
   - Always/almost always (>90% of the time)
4. Among those for whom you recommend HCV screening, how frequently do your baby boomer patients actually complete the test?
   - Never/almost never (<10% of the time)
   - Occasionally (10-39% of the time)
   - About half the time (40-59% of the time)
   - Usually (60-90% of the time)
   - Always/almost always (>90% of the time)
5. Do you have any other thoughts about HCV screening or treatment that you’d like to share with us today?

Okay now I need to ask you some questions about yourself. To see if there are common characteristics among individuals we are interviewing.

1. What is your primary clinical specialty?
2. How long have you been practicing medicine?
3. What is your age?
4. What is your race?
5. Do you consider yourself Hispanic or Latino?
6. How would you describe your gender?

Do you have any other questions or comments?
