# Supplementary material for: There’s just not enough time: a mixed methods pilot study of hepatitis C virus screening among baby boomers in primary care
Source: BMC Fam Pract. 2020 Dec 2;21:248. doi: 10.1186/s12875-020-01327-2 (PMC7713319; doi:10.1186/s12875-020-01327-2)
Supplement: Supplementary file 2 — Additional file 2. [file 12875_2020_1327_MOESM2_ESM.docx]

**General HCV questions**

1. Which of the following best describes your personal practice relating to HCV screening? *(mark all that apply)*

 I rarely screen patients for HCV infection

 I screen patients who have behavioral risk factors (e.g. injection drug use)

 I screen patients when clinically indicated (e.g. elevated ALT)

 I screen patients who have age-based risk factors (e.g. born between 1945-1965)

1. Are you aware of the birth cohort CDC recommendations for HCV screening?

□ Yes, my practice follows these recommendations

□ Yes, but we have not integrated these into practice

□ No, not aware

1. **Which of the following are prompts that you use to help remember to recommend HCV screening to your patients? (mark all that apply):**

 EMR-based reminders (e.g. flags, pop-up messages, best practice alerts, etc.)

 Pre-visit planning with the health care team

 Reminders from nurses or other health care team members prior to seeing the patient

 None of the above

 Other (please specify_______________________)

**HCV Recommendation**

1. In the past 12 months, how often did you *recommend* HCV screening to your baby boomer patients?

□ Never/almost never (approximately 10% of the time)

□ Occasionally (approximately 10-39% of the time)

□ About half of the time (approximately 40-59% of the time)

□ Usually (approximately 60-90% of the time)

□ Always/almost always (greater than 90% of the time)

□ I do not see baby boomer patients

1. How *strongly* do you recommend HCV to your baby boomer patients?

□ I strongly recommend

□ I recommend, but not strongly

□ I make no recommendation for or against HCV screening in this group

□ I recommend against

□ I do not see baby boomer patients

1. How do you usually present HCV screening to your baby boomer patients?

□ HCV screening is routine

□ HCV screening is optional

□ I do not discuss HCV screening with baby boomers

□ I do not see baby boomer patients

Barriers to Screening/Treatment

1. **Please indicate the degree to which you agree or disagree with the following statements regarding your baby boomer patients:**

|  | **Strongly Disagree** | **Disagree** | **Neither Disagree nor Agree** | **Agree** | **Strongly Agree** |
| --- | --- | --- | --- | --- | --- |
| I do not have time to discuss HCV screening with my patients | □ | □ | □ | □ | □ |
| I am not comfortable managing my patients if they screen positive for HCV infection. | □ | □ | □ | □ | □ |
| Screening for HCV infection is a less-urgent problem for my patients, compared to their other problems | □ | □ | □ | □ | □ |
| My patients are not interested in screening when I recommend it for them | □ | □ | □ | □ | □ |
| My patients do not have insurance to cover the cost of HCV screening | □ | □ | □ | □ | □ |
| The cost for HCV treatment is a barrier for my patients | □ | □ | □ | □ | □ |
| Please list any other barriers your patients experience that are not already listed in this table: |  | | | | |

Provider Self-efficacy

1. **How would you rate your proficiency in the following areas, specifically in regards to your baby boomer patients?**

|  | **None** | **Limited knowledge/skills** | **Average among my peers** | **Very knowledgeable/**  **Skilled** | **Expert- can teach others** |
| --- | --- | --- | --- | --- | --- |
| Ability to identify patients who should be screened for HCV | □ | □ | □ | □ | □ |
| Ability to discuss HCV infection and screening with patients | □ | □ | □ | □ | □ |
| Ability to adequately refer patients to the proper specialist for care | □ | □ | □ | □ | □ |
| Ability to execute the proper next steps should a patient screen positive for HCV antibody. | □ | □ | □ | □ | □ |
| Ability to treat HCV-infected patients and manage side effects | □ | □ | □ | □ | □ |
| Ability to provide a brief alcohol screen, counseling, and referral for alcohol use treatment services as needed | □ | □ | □ | □ | □ |
| Ability to assess and manage substance abuse comorbidities in patients with HCV infection | □ | □ | □ | □ | □ |
| Ability to implement in-clinic procedures for universal screening of baby boomers | □ | □ | □ | □ | □ |

HCV Information

1. Please indicate whether you agree, disagree, or are unsure about the following statements:

|  | **Agree** | **Disagree** | **Unsure** |
| --- | --- | --- | --- |
| If someone is infected with HCV, they will most likely carry the virus all their lives | □ | □ | □ |
| You can get HCV by getting a blood transfusion from an infected donor | □ | □ | □ |
| You can get HCV by having sex with someone who has HCV | □ | □ | □ |
| Perinatal transmission of HCV is not possible and an infected mother cannot pass it to her child. | □ | □ | □ |
| HCV can be transmitted through contact with contaminated needles or sharp instruments with HCV-infected blood on them. | □ | □ | □ |
| You can contract HCV by injection drug use, even if only a few times | □ | □ | □ |
| People who report risk behaviors for HCV infection should get screened yearly | □ | □ | □ |
| The CDC and USPSTF recommend universal HCV screening for baby boomers *only if* they report a behavioral risk factor | □ | □ | □ |
| Available curative treatments for chronic HCV infection have substantial side effects | □ | □ | □ |
| Approximately 1 in 30 baby boomers is currently infected with HCV | □ | □ | □ |

**Practice characteristics**

***The following questions are about your primary clinical practice:***

1. **Which of the following describes your *primary* clinical specialty? (*please* *choose one*)**

□ Family Medicine

□ Internal Medicine

□ Other

If other, please specify: _________________________________________

1. How many years have you been practicing medicine? ___________________
2. Including you, how many physicians are at the site where you usually practice?

□ 1 □ 2-5 □ 6-15 □ 16-49 □ 50-99 □ 100+

1. What percentage of your professional activity is direct patient contact?

□ Less than 25% □ 26-50% □ 51-75% □ More than 75%

1. What is the racial/ethnic category that best describes the majority of the patients (i.e., >50%) in your primary practice? (*please choose one response*)

□ Non-Hispanic White

□ Non-Hispanic Black

□ Hispanic

□ Native American/Alaska Native

□ Asian

□ Native Hawaiian/Pacific Islander

□ Other (including multiracial)

□ No definable racial/ethnic majority

1. For your primary clinical site, what proportion of your patients are baby boomers?

□ 0-10%

□ 11-25%

□ 26-50%

□ 51-75%

□ 76-100%

□ Unsure

1. **What category best describes the primary payment method for the majority of your patients (i.e., >50%) in your primary practice? (*please choose one response)***

 Employer provided private insurance (e.g. HMO, PPO, POS, etc.)

 Individually purchased private insurance (i.e. through the Health Insurance Marketplace [Healthcare.gov])

 Medicaid

 Medicare

 Other Public Insurance (e.g. state sponsored plan, etc.)

 Military Insurance (TRICARE, VA, CHAMP-VA, etc.)

 Uninsured/self-pay

 Other

1. Does your clinic receive Ryan White funding?

 Yes

 No

 I don’t know

1. On a typical day in your primary practice, about how many patients do you see? *(please choose one response)*

□ Less than 15

□ 15 to 19

□ 20 to 24

□ 25 to 29

□ 30 or more

**Provider demographic characteristics**

***The remaining questions are asked so that we know the characteristics of physicians reached***

***by this survey.***

30. What is your age? ________

31. Are you:

□ Male □ Female

1. Which term best describes your race/ethnic group? *(please choose one response)*

□ White/Caucasian □ Black/African-American

□ Asian □American Indian/Alaska Native

□ Native Hawaiian/Pacific Islander □ Mixed race

□ Other □ Prefer not to answer

1. **Are you Hispanic or Latino?**

□ Yes □ No
